# Influenza and schizophrenia: How can we shed a light in the new virus from an old association?

**DOI:** 10.1192/j.eurpsy.2021.447

**Published:** 2021-08-13

**Authors:** D. Magalhães, F. Ferreira, T. Ferreira, I. Figueiredo, F. Martinho, R. Felício, N. Santos

**Affiliations:** 1 Psychiatry, Hospital Professor Doutor Fernando Fonseca, Lisboa (Amadora), Portugal; 2 Mental Health Department, Hospital Professor Doutor Fernando Fonseca, Lisboa (Amadora), Portugal

**Keywords:** schizophrénia, influenza, viral, infection

## Abstract

**Introduction:**

COVID-19 raises serious concerns regarding its unknown consequences for health, including psychiatric long term outcomes. Historically, influenza virus has been responsible for pandemics associated with schizophrenia. Epidemiological studies showed increased risk for schizophrenia in children of mothers exposed to the 1957 influenza A2 pandemic. Controversy remains concerning the mechanisms of pathogenesis underlying this risk.

**Objectives:**

We aim to review the evidence for the association between influenza infection and schizophrenia risk, the possible pathogenic mechanisms underlying and correlate these findings with the schizophrenia hypothesis of neurodevelopment.

**Methods:**

We reviewed literature regarding evidence from epidemiological, translational animal models and serological studies using medline database.

**Results:**

The biological mechanisms likely to be relevant account to the effects of infection-induced maternal immune activation, microglial activation, infection-induced neuronal autoimmunity, molecular mimicry of the influenza virus, neuronal surface autoantibodies and psychosis with potential infectious antecedents. Influenza infection may fit into the theory of the neurodevelopment of schizophrenia as a factor that alters the normal maturation processes of the brain (possible second or third hit).

**Conclusions:**

Influenza infection has multiple pathogenic pathways in both pre and post natal processes that might increase the risk of schizophrenia or psychosis. The existing evidence regarding the relationship between influenza virus and psychosis might help us draw similar long-term concerns of COVID-19.

**Disclosure:**

No significant relationships.

